# One-Step Synthesis of Ultrathin Zeolitic Imidazole Framework-8 (ZIF-8) Membrane on Unmodified Porous Support via Electrophoretic Deposition

**DOI:** 10.3390/membranes12111062

**Published:** 2022-10-28

**Authors:** Yufan Ji, Yuyang Song, Yiping Huang, Hao Zhu, Changhai Yue, Fujian Liu, Jing Zhao

**Affiliations:** 1 China Construction Industrial & Energy Engineering Group, Nanjing 210023, China; 2 State Key Laboratory of Materials-Oriented Chemical Engineering, College of Chemical Engineering, Nanjing Tech University, Nanjing 211816, China

**Keywords:** ZIF-8, membrane, ultrathin, electrophoretic deposition

## Abstract

Metal–organic frameworks (MOFs) are regarded as the next-generation, disruptive membrane materials, yet the straightforward fabrication of ultrathin MOF membranes on an unmodified porous support remains a critical challenge. In this work, we proposed a facile, one-step electrophoretic deposition (EPD) method for the growth of ultrathin zeolitic imidazole framework-8 (ZIF-8) membranes on a bare porous support. The crystallinity, morphology and coverage of ZIF-8 particles on support surface can be optimized via regulating EPD parameters, yet it is still difficult to ensure the integrity of a ZIF-8 membrane with the constant voltage mode. In contrast, the constant current mode is more beneficial to the growth of a defect-free ZIF-8 membrane due to the steady migration rate of colloid particles toward the electrode. With a current of 0.65 mA/cm^2^ and deposition time of 60 min, a 300 nm thick ZIF-8 membrane was obtained, which exhibits a CO_2_ permeance of 334 GPU and a CO_2_/CH_4_ separation factor of 8.8, evidencing the defect-free structure.

## 1. Introduction

The precise separation of gas mixtures is an energy-intensive process in industrial and environmental fields, which is mainly achieved via adsorption, absorption cryogenic distillation and membrane technologies. Among them, membrane-based gas separation technology has attracted growing research attention owing to its high potential to enhance energy efficiency and cut down the operation cost of chemical processes [1,2,3,4]. To acquire advanced membrane technology, it is essential to develop high-performance membrane materials with both high permeance and high selectivity [5,6,7,8,9]. Currently, polymers are the dominated membrane materials that are commercialized because of their excellent processability. However, the separation performance is limited by the well-known trade-off effect between permeability and selectivity [10]. To overcome this issue, many novel advanced materials including zeolite, metal–organic frameworks (MOFs) and ionic liquids (ILs) are proposed as candidate membrane materials because they can simultaneously achieve high permeability and high selectivity [11,12,13]. ILs-based membranes possess highly tunable chemical structures that have preferential solvation properties for many gases or vapors and thus have demonstrated excellent separation toward the separation of CO_2_ or volatile organic compounds (VOCs) [14,15]. Zeolite membranes show outstanding gas separation performance toward many gas pairs including CO_2_/CH_4_, CO_2_/N_2_ and H_2_/CO_2_ due to the well-defined rigid pores, although their scalable and economical production faces difficulties [11]. Recently, MOFs has been proposed as one of the most promising molecular sieving membrane materials for the separation of many challenging gas mixtures, including olefin/alkane, owing to their well-defined micropores with exceptionally high tunability as well as their abundant membrane-fabrication methods [16,17]. Generally, MOFs are constructed from organic linkers and metal ions or metal ion clusters, exhibiting regular crystalline lattices and ordered micropores [18]. Among the various MOFs, zeolitic imidazole framework-8 (ZIF-8) is the most widely investigated one [19]. The ZIF-8 membrane possesses several advantages: (i) the ZIF-8 membrane shows excellent thermal/chemical stability owing to the relatively strong tetracoordinated Zn-N bonds; (ii) the pore aperture of ZIF-8 is 3.4 Å, which theoretically can distinguish many industry-relevant gas pairs including CO_2_/N_2_ and CO_2_/CH_4_; (iii) ZIF-8 has outstanding thin-film processability and can be synthesized on both inorganic and polymer supports under very mild conditions using various methods [20].

ZIF-8 membranes are generally fabricated by coating seeding layers followed by secondary growth to close the gaps between seeds [21]. To advance the development of ZIF-8 membranes to achieve higher performance, various methods have been developed including contra-diffusion, interfacial polymerization, and chemical vapor deposition [22,23]. However, it still remains difficult to fabricate ultrathin ZIF-8 membranes on porous supports using a straightforward method. 

Electrophoretic deposition (EPD) is a widely used film deposition technology, in which the charged particles in suspension move toward the anode or cathode under a direct current electric field and then deposit onto the electrode surface [24,25,26,27]. With regard to the synthesis of the ZIF-8 membrane, the positively charged ZIF-8 nuclei can be facilely accumulated near the cathode surface, so as to confine the subsequent nucleation, crystallization and crystal growth in a narrow region, and facilitate the formation of an ultrathin and highly intergrown ZIF-8 membrane. Agrawal and coworkers reported an electrophoretic nuclei assembly for crystallization of a highly intergrown thin-films (ENACT) route to prepare ZIF-8 membranes, wherein EPD was used for seeding a 100 nm thick ZIF-8 nuclei film [24,26]. However, this ENACT method requires secondary growth to obtain a high-quality membrane. To the best of our knowledge, no one has reported the synthesis of defect-free ZIF-8 membranes directly using the EPD method. 

In this work, we fabricated a 300 nm thick ZIF-8 membrane on an unmodified porous support using a one-step EPD process within 60 min. The ZIF-8 membranes exhibit a CO_2_ permeance of 334 GPU and a CO_2_/CH_4_ selectivity of 8.8, indicating the defect-free structure. Our results demonstrate the growth of MOF membranes on a non-conducting porous substrate under an external electric field, which hold great potential for practical application.

## 2. Materials and Methods

### 2.1. Materials

Zn(NO_3_)_2_∙6H_2_O was purchased from Sigma Aldrich. 2-Methylimidazole, acetone, isopropanol and NaOH were supplied by Sinopharm Chemical Reagent Co., Ltd., Shanghai, China. All the above regents are of analytical purity. Polyacrylonitrile (PAN) substrate (MWCO: 100 kDa) was purchased from Shandong Megavision Membrane Technology & Engineering Co., Ltd., Shanghai, China. Whatman anodized aluminum (AAO) substrate (pore size: 0.1 μm, diameter: 13 mm) was purchased from Shanghai Tianbang Industrial Co., Ltd., Shanghai, China. Both the PAN and AAO supports are of flat-sheet configuration. Copper foil (thickness: 0.127 mm, 99.9%) was supplied by Beijing InnoChem Science & Technology Co., Ltd., Beijing, China. Deionized water was homemade in the laboratory.

### 2.2. Preparation of ZIF-8 Membranes

Two pieces of copper foil with a size of 3 × 1.2 cm^2^ were utilized as the electrodes. Before performing EPD, the copper foils were washed by isopropanol and acetone under ultrasonic treatment. Then, PAN or AAO substrate was attached to one piece of copper foil (cathode).

**Figure 1:**
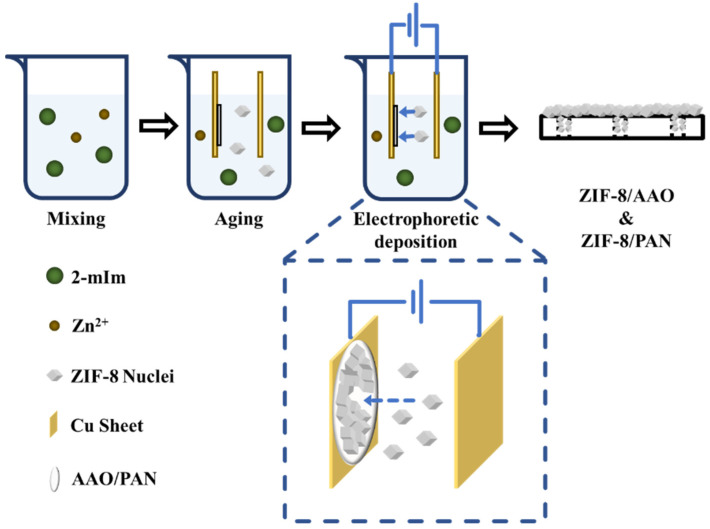
Schematic diagram of the preparation process of ZIF-8 membrane with electrophoretic deposition method (2-mIm: 2-methyimidazole).

The fabrication of ZIF-8 membranes on a substrate was conducted as follows (Figure 1). First, a ZnNO_3_ solution with a concentration of 18.5 mmol/L and a 2-methyimidazole with a concentration of 1.38 mol/L using DI water as the solvent were prepared. Second, 30 mL of ZnNO_3_ aqueous solution was mixed with a certain amount of 2-methyimidazole solution by stirring for 30 s. Third, the cathode copper foil attached with a substrate and the anode copper foil were immersed into the mixture solution. The electrodes were connected with an electrochemical station, and the distance between the electrodes was set as 1 cm. Fourth, the EPD process was started after aging the precursor solution for 3 min. After a certain time, the EPD process was stopped by switching off the electric current. The membranes were taken off from the copper foil and placed in a petri dish for drying at room temperature. The resulting membranes using PAN and AAO as supports were denoted as ZIF-8/PAN and ZIF-8/AAO, respectively. 

### 2.3. Characterizations

X-Ray diffraction (XRD, Rigaku Miniflex 600X, Tokyo, Japan) was used to characterize the crystalline structure of the membrane materials in the range of 5° ≤ 2θ ≤ 40° with a step width of 0.05° and a scan rate of 5 °/min. Field emission scanning electron microscopy (SEM, Hitachi S4800, Tokyo, Japan) equipped with a EDX was used to observe the surface and cross-sectional morphology of ZIF-8 membranes and the elemental distribution in the membranes. Before characterizing the membranes using SEM, the membranes were sputtered with metal for electron conduction.

### 2.4. Gas Separation Performance Test

**Figure 2:**
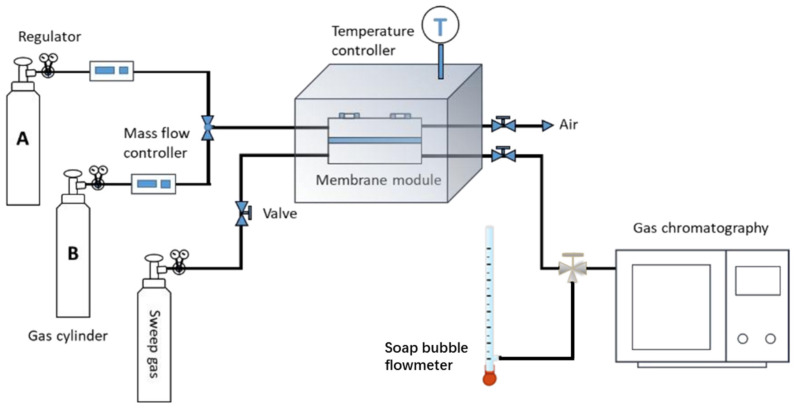
Schematic of the device for gas separation test.

The device for gas separation test mainly comprises a gas cylinder, membrane module and gas chromatography (GC, Agilent, 7820A, Santa Clara, CA, USA) as shown in Figure 2. During the test, the ZIF-8 membrane was placed in a homemade membrane module. CO_2_/CH_4_, CO_2_/N_2_ or CO_2_/H_2_ mixture (50 vol%:50 vol%) was fed into the upstream side, while argon was used as sweep gas that flowed into the downstream side. The permeate gas was brought into GC by sweep gas Ar for composition analysis, and the area ratio of the chromatographic peaks can be optimized via tuning the flow rate of Ar, to obtain more precise result. The total gas flow rate on the downstream side (sweep gas and permeate gas) can be measured by the soap bubble flowmeter. Then, the permeance of different gas components (*P/l*, GPU, Gas Permeance Unit, 1 GPU = 3.35 × 10^−10^ mol/(m^2^ s Pa)) can be calculated with Equation (1) [28,29] via combining the results from soap bubble flowmeter and GC.

(1) $$P_{i}/l=\frac{\Delta V_{i}}{\Delta t_{i}}\cdot \frac{273.15}{273.15+T}\cdot \frac{1}{1000\cdot 22.4}\cdot \frac{1}{A}\cdot \frac{1}{p_{0}x_{i}-p_{1}y_{i}}\;$$

where Δ*V*_i_/Δ*t*_i_ is the volume change rate on the downstream side (mL/s), *T* is the temperature (°C), *A* is the effective membrane area (m^2^), *p_0_* is the pressure on the feed side (100,000 Pa), *p_1_* is the total pressure on the permeate side (Pa), *x_i_* is the volume fraction of component *i* in the feed gas, and *y_i_* is the volume fraction of component *i* in the downstream side gas, which can be obtained by chromatographic analysis. 

The separation factor of the two components in the gas mixture (*α_i/j_*) can be calculated by Equation (2).

(2) $$\;\alpha_{i/j}=\frac{y_{i}/y_{j}}{x_{i}/x_{j}}$$

where *i* and *j* represent different components, and x and y represent the volume fraction of each component in the feed gas and permeate gas, respectively.

## 3. Results and Discussion

The fabrication of ZIF-8 membranes on porous supports is illustrated in Figure 1. A porous support of AAO or PAN was placed between a cathode and an anode. After aging the precursor solution for 3 min, we started the EPD process by applying an electric field between the electrodes. Driven by the electric field, the ZIF-8 nuclei could move toward the cathode and deposit onto the porous support. After a certain time, a highly intergrown ZIF-8 thin-film can be formed on the porous support. Both constant voltage mode and constant current mode were utilized to establish the electric field. We found the operation mode to supply the electric field that plays an important role on the structure of the as-synthesized ZIF-8 membranes, which is discussed in detail as follows.

### 3.1. Constant Voltage Mode

#### 3.1.1. Influence of Zn^2+^/2-methyimidazole Molar Ratio

**Figure 3:**
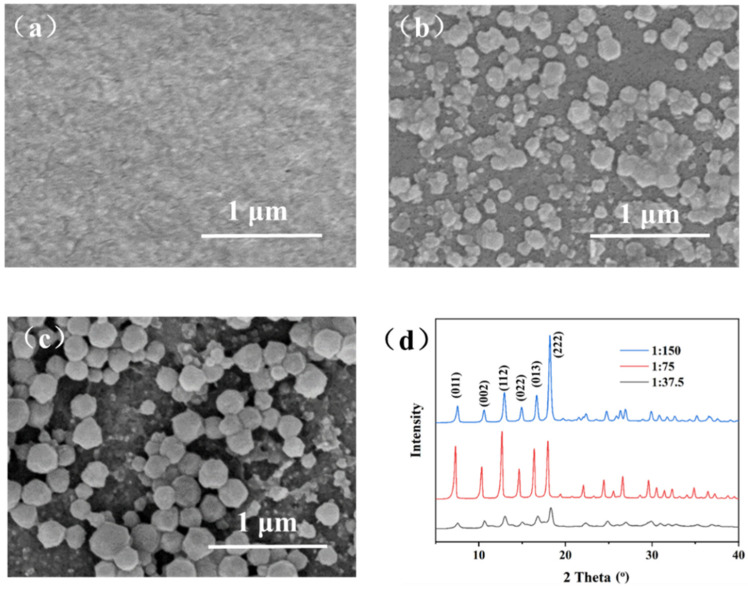
SEM images of ZIF-8 particles on PAN support with different molar ratios of zinc ions to 2-methylimidazole (constant voltage: 1 V, deposition time: 10 min): (**a**) 1:37.5, (**b**) 1:75, (**c**) 1:150; (**d**) XRD patterns of ZIF-8 particles.

The influence of the Zn^2+^/2-methyimidazole molar ratio on the ZIF-8 membrane structure was studied with a constant voltage of 1 V and deposition time of 10 min. The Zn^2+^/2-methyimidazole molar ratios are 1:37.5, 1:75, and 1:150. When the molar ratio is 1:37.5, we cannot observe any particles on the surface of the support (Figure 3a), indicating that ZIF-8 was not synthesized or that the ZIF-8 particles are too small to be detected by SEM. When the molar ratio is 1:75 or 1:150 (Figure 3b,c), sub-micrometer-scale particles appear on the surface of the support, indicating the formation of ZIF-8. However, the intergrown ZIF-8 film has not been formed under such conditions, very likely because the deposition time is not sufficient. The formation of ZIF-8 was confirmed by XRD patterns (Figure 3d). When the molar ratio is 1:37.5, the characteristic peak of ZIF-8 is very weak, indicating that the coverage of ZIF-8 on the support surface is very small, consistent with the SEM image. When the molar ratio reduces to 1:75, extremely sharp XRD peaks are observed from the corresponding product, evidencing the high crystallinity. When the molar ratio further reduces to 1:150, the product also shows sharp XRD peaks, but the crystal size is increased to about 230 nm, which is much bigger than that using the molar ratio of 1:75. The big crystal size is not beneficial for fabricating ultrathin ZIF-8 membranes. Overall, the Zn^2+^/2-methyimidazole molar ratio of 1:75 is optimal for the fabrication of ultrathin ZIF-8 membranes, and therefore, such a molar ratio of Zn^2+^/2-methyimidazole was utilized for achieving ultrathin ZIF-8 membranes in the following studies.

#### 3.1.2. Influence of EPD Voltage

**Figure 4:**
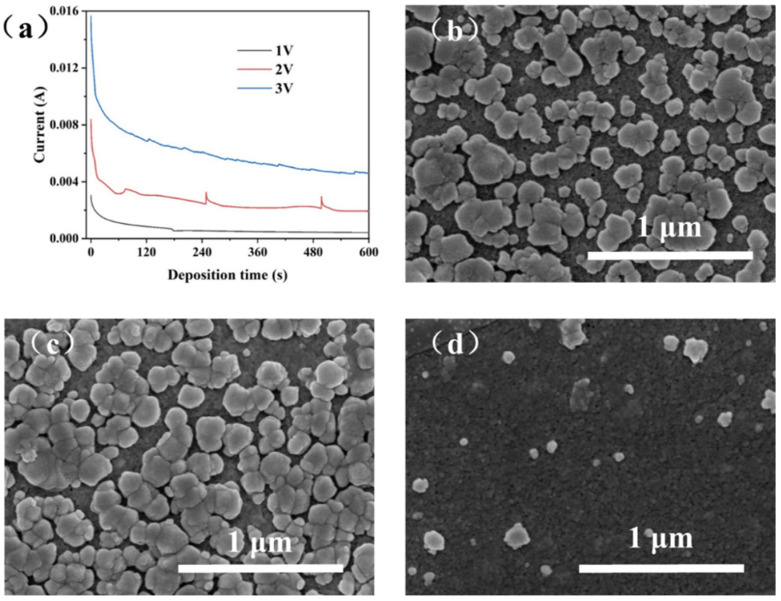
(**a**) The current–time curve under different constant voltages; SEM images of ZIF-8 particles on support: (**b**) 1 V, (**c**) 2 V, (**d**) 3 V (molar ratio of zinc ion to 2-methylimidazole: 1:75, deposition time: 10 min).

Under an external electric field, the charged ZIF-8 nuclei particles mobilize toward the cathode with a flux that is proportional to the strength of the applied electric field [30,31,32]. Thus, the applied voltage strongly affects the transfer rate of ZIF-8 nuclei to the porous support as well as the crystal growth process. We plotted the curves of electric current as a function of the deposition time for the EPD processes with a voltage of 1, 2, or 3 V and a deposition time of 10 min, as displayed in Figure 4a. The higher EPD voltage leads to higher current because of the higher transfer rate of the charged ZIF-8 nuclei. With increasing the deposition time, the current decreases rapidly in the initial stage of 1–60 s and then decreases slowly in the subsequent time. Such a phenomenon can be attributed to the fact that in the initial stage, the charged ZIF-8 nuclei are moved toward the surface of porous PAN surface, leading to the coverage of PAN support by the non-conducting ZIF-8 nuclei. The deposition of ZIF-8 on the PAN surface significantly increases the electric resistance between the electrodes, thus reducing the current as well as the deposition rate of the ZIF-8 nuclei.

Figure 4b-d show the surface morphology of the PAN support after the EPD process using different voltages. It can be observed that when the applied voltage is 3 V, nearly no ZIF-8 particles are deposited onto the PAN surface. This phenomenon is attributed to the rapid water dissociation into H_2_ and the generation of numerous bubbles near the cathode at high voltage, which can subsequently prevent the attachment of ZIF-8 nuclei on the PAN surface. When the applied voltage is 2 or 1 V, the PAN surface is covered with abundant ZIF-8 crystals with high coverage, which is beneficial for the growth of a highly intergrown ZIF-8 film. However, at a voltage of 2 V, water dissociation still occurs, leading to the occasional peeling off of ZIF-8 particles attached on the support and the suddenly increased current (Figure 4a). The similar phenomenon of current increment also appears at the voltage of 3 V, wherein the less particles on the support surface result in the weaker fluctuation of current. Due to the above reasons, the deposition voltage of 1 V was adopted in the following EPD processes. 

#### 3.1.3. Influence of Deposition Time

**Figure 5:**
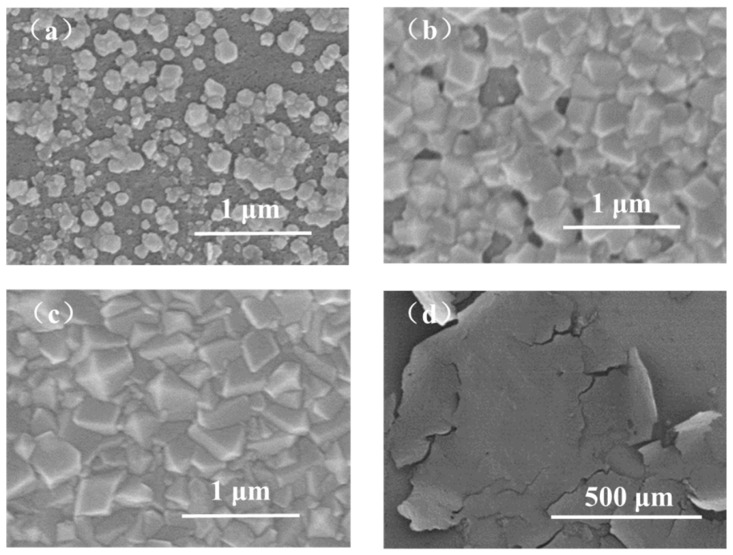
SEM images of ZIF-8/PAN membranes prepared with different deposition times: (**a**) 10 min; (**b**) 40 min; (**c**,**d**) 60 min (constant voltage: 1 V, molar ratio of zinc ion to 2-methylimidazole: 1:75).

To achieve the growth of a highly intergrown ZIF-8 membrane on the support, we investigated the growth of ZIF-8 membranes as a function of EPD time, and the surface morphologies are shown in Figure 5. When the EPD time is 10 min, only a small fraction of the porous support is covered by ZIF-8 crystals, and most of the ZIF-8 crystals are not intergrown. When the EPD time is extended to 40 min, an intergrown ZIF-8 membrane is obtained. However, many defects or pinholes can be observed from the surface morphology, wherein the uncovered area approximately accounts for 5% of the total surface area. When the EPD time is further extended to 60 min, the ZIF-8 membrane becomes highly intergrown and shows a characteristic rhombic dodecahedron morphology (Figure 5c). However, many cracks or even peeling-off of the ZIF-8 film can be observed when increasing the scanning area of the SEM by lowering the magnification time. This phenomenon is very likely caused by the weak interactions between the ZIF-8 film and PAN support. Moreover, the flexible PAN support also makes the generation of cracks easier during the handling of the support [33]. 

**Figure 6:**
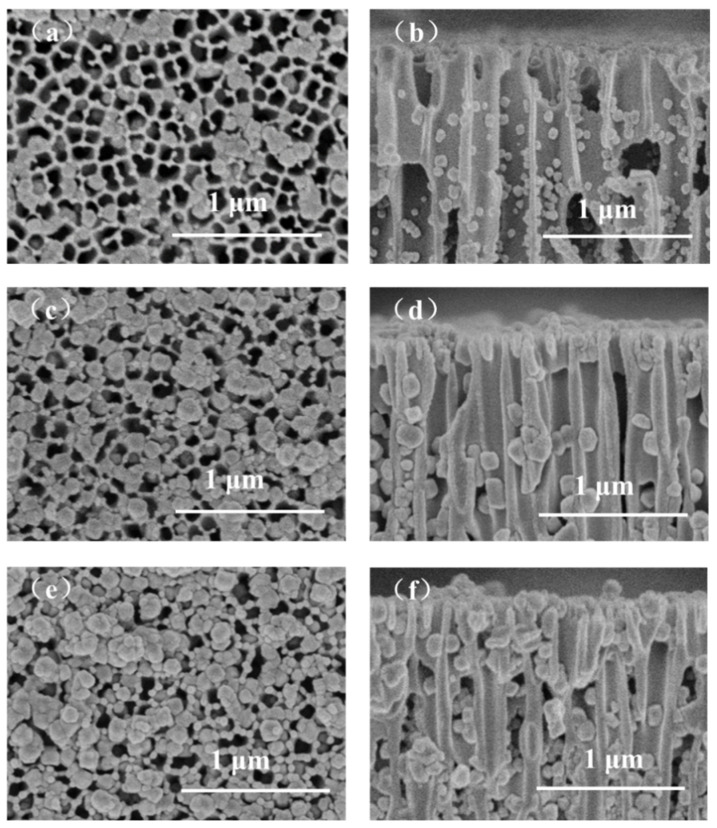
SEM images of ZIF-8/AAO membranes prepared with different deposition times, in which the left images are membrane surfaces and the right images are membrane cross-sections: (**a**,**b**) 20 min, (**c**,**d**) 40 min, (**e**,**f**) 60 min (constant voltage: 1 V, molar ratio of zinc ion to 2-methylimidazole: 1:75).

We also tried to utilize a rigid AAO support to replace PAN for EPD of the ZIF-8 membranes, and the morphologies of the resulting AAO supported ZIF-8 are presented in Figure 6. With the increase in deposition time, it can be observed that the loading of ZIF-8 particles onto the surface or in the channels of AAO is increasing, and the particle size becomes larger. However, even when the deposition time is extended to 60 min, we could not obtain defect-free ZIF-8 membranes, and a large fraction of AAO surface is still not covered by ZIF-8 particles. Such a phenomenon is very likely attributed to the fact that the AAO support contains much larger pore size than the PAN support and the pore area density of AAO is also higher than the PAN support. In such a case, the ZIF-8 particles can more easily move into the AAO channels, and the chance of growing on the AAO surface becomes much lower. Moreover, the utilization of constant voltage mode for the EPD process may be not beneficial for the growth of highly intergrown and full-coverage ZIF-8 membranes. Under constant voltage mode, with the increase in ZIF-8 particle loading in AAO channels and on the AAO surface, the electric resistance would increase, which can decrease the electric current and the mobility of ZIF-8 particles toward the AAO support. Therefore, it is difficult to obtain a defect-free ZIF-8 membrane on the AAO support with constant voltage mode.

### 3.2. Constant Current Mode

#### 3.2.1. Influence of Electric Current

**Figure 7:**
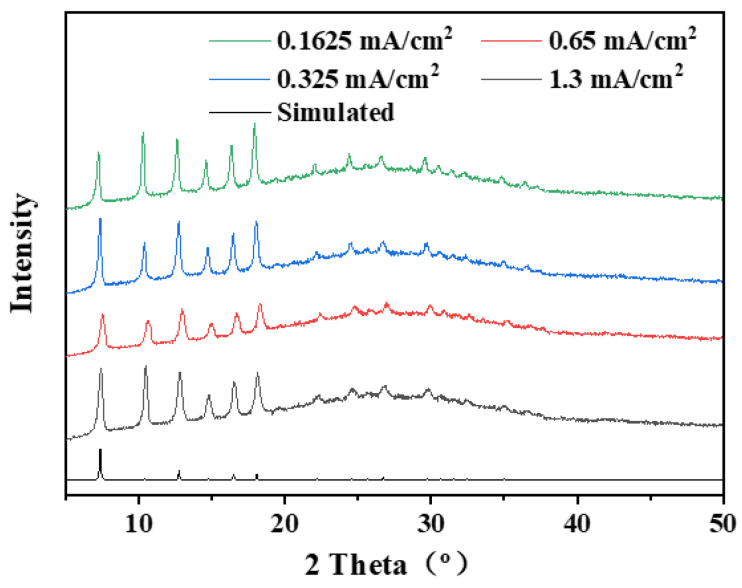
XRD patterns of ZIF-8/AAO membranes deposited for 60 min under different deposition currents.

**Figure 8:**
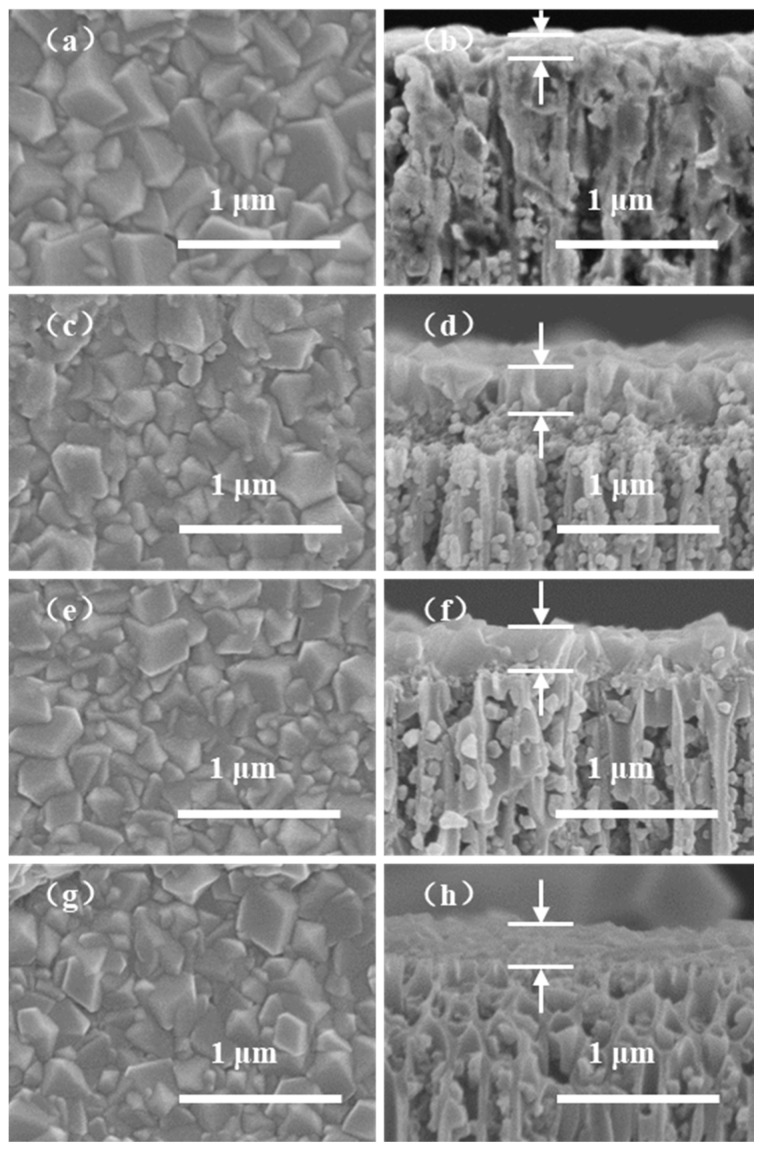
SEM images of ZIF-8/AAO membranes deposited for 60 min under different deposition currents, in which the left images show the surface morphology and the right images show the cross-section morphology: (**a**,**b**) 0.1625 mA/cm^2^, (**c**,**d**) 0.325 mA/cm^2^, (**e**,**f**) 0.65 mA/cm^2^, (**g**,**h**) 1.3 mA/cm^2^.

Compared with the constant voltage mode, the constant current mode can ensure the migration rate of colloid particle constants, benefiting the growth of defect-free ZIF-8 membranes [17,34]. We fabricated four types of AAO-supported ZIF-8 membranes using an EPD time of 60 min, and the corresponding applied currents are 0.1625, 0.325, 0.65, 1.3 mA/cm^2^, respectively. The resulting ZIF-8/AAO membranes were characterized by XRD and SEM, as shown in Figure 7 and Figure 8. All the ZIF-8 membranes present sharp characteristic peaks in XRD, indicating the high crystallinity of the ZIF-8 membranes. The surface and cross-section morphologies of the ZIF-8 membranes show that all the ZIF-8 membranes present characteristic rhombic dodecahedron morphology with defect-free or pin-hole-free features (Figure 8). The ZIF-8 membrane thickness is 150 nm when a current of 0.1625 mA/cm^2^ is used. When increasing the applied current, the membrane thickness increases owing to the enhancement of migration rate of ZIF-8 particles. The ZIF-8 membranes will be further investigated by gas permeation measurement to indicate whether minor defects are present in the membranes, as discussed in the next section.

#### 3.2.2. Influence of EPD Time

**Figure 9:**
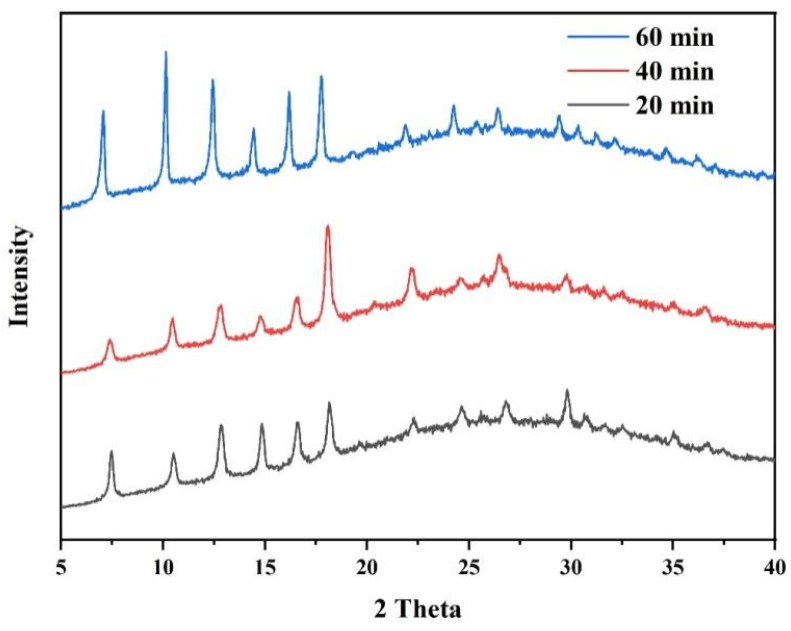
XRD patterns of ZIF-8/AAO membranes with different deposition times.

We tracked down the deposition of ZIF-8 membranes with different growth times (20, 40, and 60 min) under an external current of 0.65 mA/cm^2^. The resulting ZIF-8/AAO membranes are characterized by XRD and SEM. The XRD patterns demonstrate that all the ZIF-8 membranes are highly crystalline, and the ZIF-8 membrane with a growth time of 60 min shows the best crystallinity (Figure 9).

**Figure 10:**
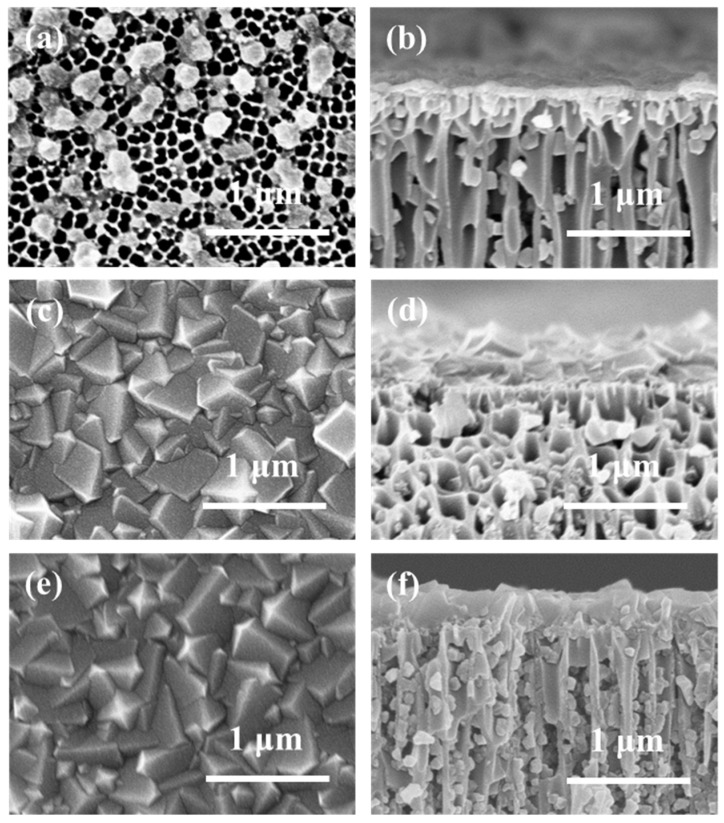
SEM images of ZIF-8/AAO membranes with different deposition times, in which the left images show the surface morphology and the right images show the cross-section morphology: (**a**,**b**) 20 min, (**c**,**d**) 40 min, (**e**,**f**) 60 min (deposition current: 0.65 mA/cm^2^).

The SEM images in Figure 10 show that when the EPD time is 20 min, only a small fraction of the AAO surface is covered by ZIF-8 crystals, indicating that 20 min is too short to deposit a highly intergrown ZIF-8 membranes. With extending the EPD time to 40 or 60 min, both the ZIF-8 membranes are highly intergrown with characteristic rhombic dodecahedron morphology. The ZIF-8 membrane thickness increases to 300 nm with an EPD time of 60 min.

**Figure 11:**
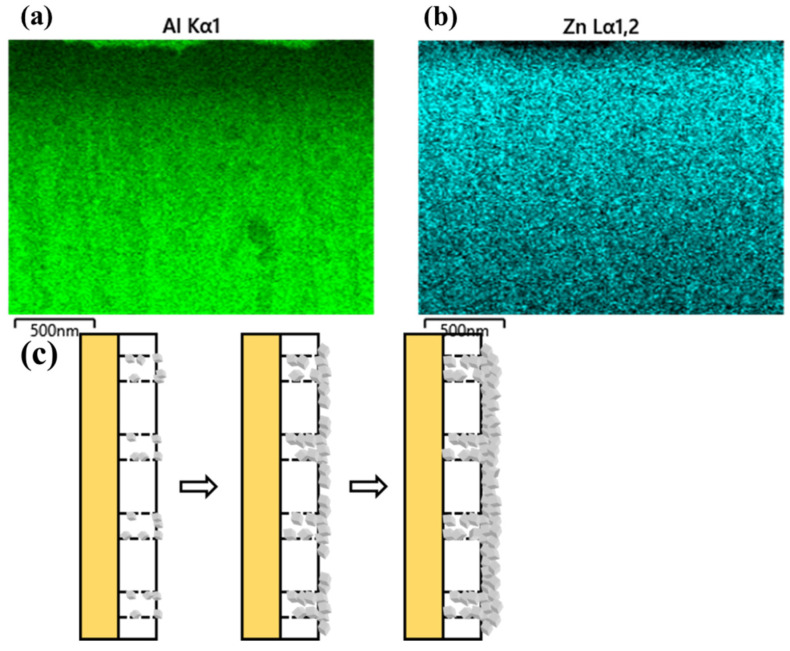
(**a**,**b**) Mapping characterization results of ZIF-8/AAO membrane prepared with deposition time of 60 min and current of 0.65 mA/cm2. (**c**) Schematic diagram of the evolution of the ZIF-8 membrane structure during formation.

The ZIF-8/AAO membrane with a growth time of 60 min under a current of 0.65 mA/cm^2^ is characterized by EDS mapping, as shown in Figure 11. The distribution of the Al element in the cross-sectional morphology of the ZIF-8/AAO membrane indicates that the membrane thickness is 300 nm, consistent with the result obtained from the SEM image. The distribution of the Zn element arising from ZIF-8 indicates that the ZIF-8 particles deposit both on the AAO surface and inside the AAO channels under the external current. The ZIF-8 deposition process is illustrated in Figure 11c. During formation of the ZIF-8 membrane, the Zn^2+^ and ZIF-8 crystal nuclei in solution move toward the cathode under the external electric field. Since the copper cathode is located on the backside of the support, the electric field formed between the cathode and anode passes through the support channels. The pore size of the AAO support is about 100 nm, which is much larger than the size of crystal nuclei (<20 nm). Therefore, a large number of crystal nuclei penetrate deep into the support pores under the force of the electric field, and then they crystallize and grow in it, leading to the low surface coverage of the AAO support at the initial stage of deposition. With the progress of electrophoretic deposition, after the pores are gradually blocked, ZIF-8 starts to deposit onto the surface of the support, and the crystals gradually become highly intergrown, finally forming a dense and defect-free ZIF-8 membrane.

### 3.3. Gas Separation Performance

Although the ZIF-8 membranes with different deposition currents all show pinhole-free morphologies (Figure 8), only the membrane prepared under a current of 0.65 mA/cm^2^ achieves effective CO_2_/CH_4_ separation with a separation factor of 8.8. With lower current, the deposition rate is slower, which leads to a thinner membrane and unsealed defects between the crystals. With higher current, there may be a few bubbles (H_2_) and a high concentration of OH^−^ near cathode due to water dissociation, which will disrupt the ZIF-8 deposition process and even corrode the already deposited ZIF-8 crystals, leading to internal membrane defects.

**Figure 12:**
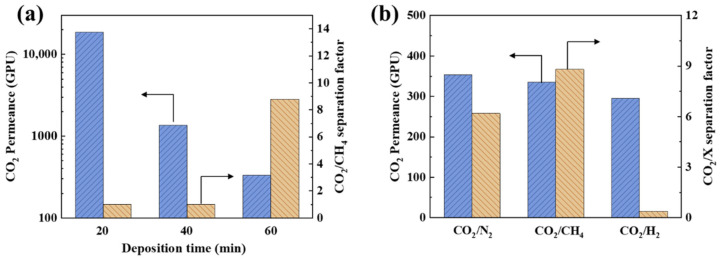
(**a**) Gas separation performance of ZIF-8/AAO membranes with different deposition times (deposition current: 0.65 mA/cm^2^). (**b**) Gas separation performance of the ZIF-8/AAO membrane for different gas mixtures (deposition current: 0.65 mA/cm^2^, deposition time: 60 min).

The CO_2_/CH_4_ mixture separation performance of the ZIF-8 membranes as a function of EPD time (with the current of 0.65 mA/cm^2^) is presented in Figure 12a. With increasing the deposition time, the CO_2_ permeance decreases from 18697 to 334 GPU. When the deposition time increases from 20 to 40 min, the CO_2_ permeance decreases drastically from 18697 to 1358 GPU while the separation factor is still about 1, indicating that the coverage of ZIF-8 particles on the AAO support increases significantly, which is in agreement with the SEM results (Figure 10). However, there are still many non-selective defects in the membrane. When the deposition time further increases to 60 min, the decrease in CO_2_ permeance becomes slow, reducing from 1358 to 334 GPU, but the separation factor rapidly increases from 1 to 8.8. With increasing the deposition time from 40 to 60 min, the surface morphology of the ZIF-8 membranes nearly changes. Both of the ZIF-8 membranes are highly intergrown without pinholes. The decrease in gas permeance and increase in gas selectivity indicate the healing of micro-defects in the ZIF-8 membranes during the growth stage from 40 to 60 min.

The gas mixture separation performance (CO_2_/N_2_, CO_2_/CH_4_ and CO_2_/H_2_) of the ZIF-8/AAO membranes was investigated with a current of 0.65 mA/cm^2^ and a deposition time of 60 min (Figure 12b). The gas mixture separation factor increases in the following trend: CO_2_/H_2_< CO_2_/N_2_< CO_2_/CH_4_. The kinetic diameter of H_2_, CO_2_, N_2_, CH_4_ are 0.29, 0.33, 0.36 and 0.38 nm, respectively, and the gas condensability increases in the following trend: H_2_< N_2_< CH_4_< CO_2_. Thus, the high CO_2_/CH_4_ and CO_2_/N_2_ selectivity could be attributed to the molecular sieving ability (size cut-off) of the ZIF-8 aperture pores, and the diffusion selectivity plays a dominating role. The H_2_ permeance is 768 GPU, which is higher than the CO_2_ permeance due to the smaller size of H_2_. The CO_2_ permeance from the H_2_/CO_2_ mixture measurement is smaller than that from the CO_2_/CH_4_ mixture, possibly attributing to the higher H_2_ diffusion coefficient, and the occupation of the channels by H_2_ can hinder the diffusion of CO_2_. Overall, we successfully achieved the deposition of 300 nm thick, defect-free ZIF-8 membranes on a non-conducting support using a one-step EPD process, and the ZIF-8 membranes demonstrated high CO_2_/CH_4_ separation performance [35,36,37].

## 4. Conclusions

In this study, we achieved the deposition of ZIF-8 membranes on a non-conductive and unmodified support with a one-step electrophoretic deposition method for the first time. The thickness and crystal defects in the ZIF-8 membranes can be easily controlled by regulating the formulation, deposition time, EPD mode (constant voltage or constant current), and voltage/current. Comparatively, the constant current mode is more desirable for the formation of a defect-free ZIF-8 membrane. The optimal deposition current is ascertained to be 0.65 mA/cm^2^, which confers an adequate deposition rate and meanwhile avoids water dissociation near the cathode. The minimal deposition time is 60 min, wherein the last 20 min is a defect-sealing process, leading to the sharply increased CO_2_/CH_4_ separation factor from 1 to 8.8. The thickness of the optimized ZIF-8 membrane is around 300 nm, which is one of the thinnest MOF membranes reported to date. As a result, the ZIF-8 membranes exhibit a CO_2_ permeance of 334 GPU with a CO_2_/CH_4_ separation factor of 8.8. Our results give insight into the process of electric-field-assisted deposition of MOF membranes and may open a new avenue for the fabrication of ultrathin, high-performance MOF membranes on non-conducting support.

## Data Availability

The data presented in this study are available on request from the corresponding author.
